# Methylation-level inferences and detection of differential methylation with MeDIP-seq data

**DOI:** 10.1371/journal.pone.0201586

**Published:** 2018-08-07

**Authors:** Yan Zhou, Jiadi Zhu, Mingtao Zhao, Baoxue Zhang, Chunfu Jiang, Xiyan Yang

**Affiliations:** 1 College of Mathematics and Statistics, Institute of Statistical Sciences, Shenzhen University, Shenzhen, China; 2 Institute of Statistics and Applied Mathematics, Anhui University of Finance & Economics, Bengbu, Anhui, China; 3 School of Statistics, Capital University of Economics and Business, Beijing, China; 4 School of Financial Mathematics and Statistics, Guangdong University of Finance, Guangzhou, China; Chuo University, JAPAN

## Abstract

DNA methylation is an essential epigenetic modification involved in regulating the expression of mammalian genomes. A variety of experimental approaches to generate genome-wide or whole-genome DNA methylation data have emerged in recent years. Methylated DNA immunoprecipitation followed by sequencing (MeDIP-seq) is one of the major tools used in whole-genome epigenetic studies. However, analyzing this data in terms of accuracy, sensitivity, and speed still remains an important challenge. Existing methods, such as BATMAN and MEDIPS, analyze MeDIP-seq data by dividing the whole genome into equal length windows and assume that each CpG of the same window has the same methylation level. More precise work is necessary to estimate the methylation level of each CpG site in the whole genome. In this paper, we propose a Statistical Inferences with MeDIP-seq Data (SIMD) to infer the methylation level for each CpG site. In addition, we analyze a real dataset for DNA methylation. The results show that our method displays improved precision in detecting differentially methylated CpG sites compared to the existing method. To meet the demands of the application, we have developed an R package called “SIMD”, which is freely available in https://github.com/FocusPaka/SIMD.

## 1 Introduction

Several studies have shown that methylation in DNA is highly related to diverse biological processes and that aberrant methylation results in severe effects, including different types of cancers [1, 2]. Therefore, the research on epigenetic modifications throughout the human genome is meaningful. Analyzing DNA methylation profiles is now feasible due to the development of next-generation sequencing techniques, such as MethylC-seq, MeDIP-seq, MBD-seq, and MRE-seq. In particular, bisulfite genomic DNA sequencing is the gold standard to profile genome-wide DNA methylation [3]. Although there are several approaches (such as MethylC-seq and whole-genome shotgun bisulfit sequencing (WGSBS)) that are reasonable for whole-genome analysis, it is rather expensive. MeDIP-seq can achieve nearly the same results as some more expensive approaches at a lower cost [4]. We therefore propose a method based on MeDIP-seq data to analyze methylation levels.

MeDIP [5] involves enrichment of the methylated DNA fractions immunoprecipitated by 5-methylcytosine-specific antibodies. Although MeDIP-seq cannot provide base pair-specific profiles, it reflects methylation levels by the number of immunoprecipitated DNA fragments. Compared to MeDIP-seq, which only enriches the methylated portion of the genome, we combine methylation-sensitive restriction enzyme sequencing (MRE-seq) to identify unmethylated CpGs. It utilizes methyl-sensitive restriction enzymes (MREs), such as HpaII (CĈGG), Hin6I(GĈGC), and AciI (CĈGC) to specifically identify unmethylated CpGs [6]. The integrative method improves accuracy to identify intermediate methylation regions and enables whole-genome identification for epigenetic states [7].

Several computational tools have been developed for analyzing MeDIP-seq data. BATMAN [8] defines a coupling factor to measure the varying densities of methylated CpG sites and then implements a Bayesian deconvolution strategy to infer the methylation status at each CpG site. Mattia Pelizzola [9] believes that the relationship between the MeDIP enrichment estimates and the actual methylation levels are not linear and presented MEDME, which is based on experimental and analytical methods to evaluate the actual relationship and predicted methylation levels. The MEDIPS [10] approach is similar to the former two methods and produces similar results as BATMAN with higher computational efficiency, which significantly reduces running time for processing MeDIP-seq data. To obtain more information for methylome coverage at a lower cost, R. Alan Harris [7] has proposed a strategy to combine MeDIP-seq and MRE-seq to calculate the methylation scores, which can be used to infer individual CpG methylation status. The M&M [11] algorithm is another method for analyzing integrative data, and is more accurate than other methods.

In this paper, we build a model of MeDIP-seq data based on [7] to estimate the methylation level for a single CpG site. We attempt to summarize the algorithm into a model, which will enable us to understand how to integrate the MRE-seq data. After MeDIP-seq and MRE-seq experiments, we map two kinds of short reads to the reference genome. It is known that MRE-seq short reads can be accurately mapped to the CpG site that contributes to it, but MeDIP-seq short reads cannot be. A short read of MeDIP-seq will cover one or more CpG sites (the short reads that cannot cover CpG sites will be discarded). Then, one short read in MeDIP-seq is pulled from only one CpG site or several neighboring CpG sites; however, we do not know which one. In order to identify the actual CpG sites that contribute to the short read and to obtain the actual number of short reads on each CpG site, it is necessary to build a model and provide statistical inferences for the MeDIP-seq data. After obtaining the actual number of short reads on each CpG site, we utilize them to detect differentially methylated CpG sites.

The remainder of the paper is organized as follows. In Section 2, we provide a brief description of the model and two possible cases and then propose theorems for those assumptions. In Section 3, we use an example to illustrate the SIMD method. In Section 4, we apply the proposed method to analyze a real dataset and compare it to the existing method. In Section 5, we end with a discussion and the conclusion.

## 2 Model for MeDIP-seq reads

In the MeDIP-seq experiment, genomic DNA is first isolated and sheared by sonication to short fragments of a few hundred basepairs. In this step, the DNA fragments contain both methylated fragments and unmethylated fragments. After immunoprecipitation with an antibody that can specifically bind the DNA methylation sites, the immunoprecipitated DNA fragments will almost only comprise methylated fragments and can be PCR amplified and sequenced. By aligning the MeDIP-seq short reads to the reference genome, the methylation levels of CpG sites in a region can be estimated based on the read counts in the region. This region-based method can provide insightful answers to numerous important biological questions, but it is of low resolution and cannot provide information about the methylation status of single CpG sites.

Though difficult, inferring the methylation level of single CpG sites based on MeDIP-seq data is not impossible. For example, provided that the coverage of MeDIP-seq data is sufficiently large, the methylation level of an isolated CpG site (a CpG site that is far away from other CpG sites) can be easily derived. If there are MeDIP-seq reads covering the site, these reads will not be able to cover other CpG sites, which implies that this CpG site is methylated; otherwise, this site is not methylated. When two or more CpG sites are close to each other (the distance between two neighboring CpG sites is less than the read length), an MeDIP-seq read covering one CpG site will also cover its neighboring CpG site, which makes it very difficult to determine which CpG site is methylated. However, as breakage induced by sonication is random, with sufficient sequencing coverage we may still be able to distinguish between the methylated and unmethylated CpG sites. For example, suppose that only two CpG sites are close to each other and are far away from other CpG sites. If only one of the two CpG sites is methylated, the DNA fragments obtained from the sonication in this region (the region that contains the two CpG sites) can be classified into three categories: the fragments overlapping with both CpG sites, the fragments overlapping only with the methylated CpG sites, or the fragments overlapping only with the unmethylated CpG sites. Because the fragments in the first two (the last) categories contain a methylated (unmethylated) CpG site, they can (cannot) be immunoprecipitated and sequenced. Therefore, we would obtain significantly more MeDIP-seq reads covering the methylated CpG site. Similarly, if both CpG sites are methylated, the number of reads covering both CpG sites should be roughly the same. Based on this observation, we can develop a statistical model to estimate the methylation level of single CpG sites.

Considering that a region *C* consists of *G* CpG sites, it is supposed that *R* is a random MeDIP-seq read sequenced from the region overlapping with some of the *G* CpG sites. From the MeDIP-seq experimental flow, we know that this read is sequenced because it contains at least one methylated CpG site and therefore allows an antibody to bind to its methylated CpG sites, which in turn makes it immunoprecipitated and sequenced. Let *X*_*Rj*_ = 1 (*j* = 1, ⋯, *G*) if the *j*th CpG site contributes to the sequencing of the short read *R*, or in other words, if the short read *R* contains the *j*th CpG site and an antibody binds to the *j*th CpG site, thereby allowing the immunoprecipitation and sequencing of short read *R*. Otherwise, we denote *X*_*Rj*_ = 0. Note that because *R* is a random read, *X*_*Rj*_ is a random variable taking values of {0, 1}. Assume that we have *n* short reads overlapping with the region *C*. Let *X*_*ij*_ (*i* = 1, ⋯, *n* and *j* = 1, ⋯, *G*) be the random variable as introduced above for the *i*th read. We denote *X*_*i*_ = (*X*_*i*1_, *X*_*i*2_, ⋯*X*_*ij*_, ⋯ *X*_*iG*_). We make the following assumptions about the random variables *X*_*ij*_.

**Assumption 1**. Assume that *X*_*i*1_, *X*_*i*2_, ⋯*X*_*ij*_, ⋯ *X*_*iG*_ are independent and follow two-point distribution, that is,

$$\begin{array}{c} X_{ij}∼\{\begin{array}{cc} 1 & \;\;\;q_{j}=\frac{\lambda_{j}}{1+\lambda_{j}}\\ 0 & \;\;\;1-q_{j}=\frac{1}{1+\lambda_{j}}, \end{array} \end{array}$$

where *j* = 1, 2, ⋯*G*, *i* = 1, 2, ⋯*n*, and *q*_*j*_ is the probability of the *j*th CpG site contributing to the sequencing of a short read. Note that this probability is composed of two parts: one is the probability that a short read contains the *j*th CpG site and the other is the probability that an antibody actually binds to this CpG site and thus allows immunoprecipitation and sequencing the short read.

This assumption essentially tells us that the random vectors *X*_*i*_ are independently and identically distributed (i.i.d.) and that their components are independent. The i.i.d. assumption of the random vectors *X*_*i*_ is reasonable because the short reads can be safely viewed as independently sampled with the same sampling procedure. The independence assumption of the components of *X*_*i*_ is relatively strong because if a read contains two methylated CpG sites, the antibody binding to one CpG site may influence the binding to the other CpG site.

**Assumption 2**. Assume one short read in MeDIP-seq is pulled from only one CpG site (the case is the same as Ting’s algorithm), that is, $\Sigma_{j=1}^{G}X_{ij}=1$.

**Assumption 3**. Assume one short read in MeDIP-seq is pulled from not less than one CpG site (each observed short read must be associated with not less than one CpG site on the genome), that is, $\Sigma_{j=1}^{G}X_{ij}\geq 1$.

Under the above assumptions, there are some theoretical results.

**Theorem 1**. Under **Assumptions 1** and **2**, the joint distribution of *X*_*i*1_, *X*_*i*2_, ⋯, *X*_*ij*_, ⋯, *X*_*iG*_ is a multinomial distribution with probability *P* = (*p*_1_, *p*_2_, ⋯, *p*_*j*_, ⋯, *p*_*G*_), where $p_{j}=\lambda_{j}/\Sigma_{j=1}^{G}\lambda_{j}$. That is,

$$\begin{array}{c} X_{i}|\Sigma_{j=1}^{G}X_{ij}=1∼Multinomial\left(P\right). \end{array}$$

Proof: Please see **Appendix A**.

**Theorem 2**. Under **Assumptions 1** and **3**, the joint distribution of *X*_*i*1_, *X*_*i*2_, ⋯, *X*_*ij*_, ⋯, *X*_*iG*_ is:

$$\begin{array}{c} p\left(X_{i1},\;\cdots ,\;X_{ij},\;\cdots ,\;X_{iG}|\Sigma_{j=1}^{G}X_{ij}\geq 1\right)=\frac{\prod_{j=1}^{G}q_{j}^{X_{ij}}{\left(1-q_{j}\right)}^{1-X_{ij}}}{1-\prod_{j=1}^{G}\left(1-q_{j}\right)}. \end{array}$$

Proof: Please see **Appendix B**.

The goal of the model is to compute the number of actual short reads in each CpG site, and to infer the methylation level. The short reads are indeed impacted by the CpG site. In real data, a short read covers some continuous CpG sites of all *G* CpG sites. Then, the *i*th short read is $x_{i}=(0,\cdots ,0,x_{ik_{i}},\cdots ,x_{il_{i}},0,\cdots ,0)$. The CpG sites of *k*_*i*_, ⋯, *l*_*i*_ are covered by the *i*th short read. At least one of $x_{ik_{i}},\cdots ,x_{il_{i}}$ are equal to 1, but we do not know which one. Therefore, we assume that the $x_{ik_{i}},\cdots ,x_{il_{i}}$ are missed. Then, we compute the actual short read number of each CpG site using the EM algorithm. The EM steps are presented in **Appendix C**.

## 3 An example of the model

**Fig 1:**
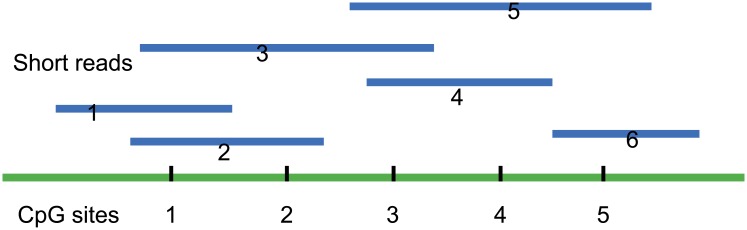
Short reads mapped to reference regions. For example, there are six short reads that cover five CpG sites.

We use a simple example to explain the model. We assume that there are five CpG sites in a region and six short reads are mapped on the region (Fig 1).

Let *X*_*ij*_ comes from a two-point distribution. That is,

$$\begin{array}{c} X_{ij}∼\{\begin{array}{cc} 1 & \;\;\;q_{j}=\frac{\lambda_{j}}{1+\lambda_{j}}\\ 0 & \;\;\;1-q_{j}=\frac{1}{1+\lambda_{j}}, \end{array} \end{array}$$ (1)

where *j* = 1, 2, ⋯, 5 and *i* = 1, 2, ⋯, 6. Therefore, the combination distribution of all CpG sites is:

$$\begin{array}{ccc} f(X_{i}) & = & f(X_{i1},X_{i2},\cdots ,X_{i5})\\ & = & \Pi_{j=1}^{5}{\left(q_{j}\right)}^{X_{ij}}{\left(1-q_{j}\right)}^{\left(1-X_{ij}\right)}\\ & = & \Pi_{j=1}^{5}{\left(\frac{\lambda_{j}}{1+\lambda_{j}}\right)}^{X_{ij}}{\left(\frac{1}{1+\lambda_{j}}\right)}^{\left(1-X_{ij}\right)}. \end{array}$$

In the model, there will be two cases.

### 3.1 Only a single CpG contributes to a short read

If one short read covers several CpG sites, it actually only comes from one of them, even though we do not know which one it is. That is, given $\Sigma_{j=1}^{5}X_{ij}=1$, the joint distribution of *X*_*i*1_, *X*_*i*2_, *X*_*i*3_, *X*_*i*4_, and *X*_*i*5_ is a multinomial distribution with probability *P* = (*p*_1_, *p*_2_, *p*_3_, *p*_4_, *p*_5_), where $p_{j}=\lambda_{j}/\Sigma_{j=1}^{5}\lambda_{j}$. A short read is an observation that is *X*_*i*_ = (*x*_*i*1_, *x*_*i*2_, *x*_*i*3_, *x*_*i*4_, *x*_*i*5_), where *x*_*ij*_ = 0 or 1. A note is that only one element of *X*_*i*_ is 1 and the others are 0. The *x*_*ij*_ will be 0 when the *j*th CpG is not covered by the *i*th short reads. That is,

$$\begin{array}{c} X_{i}|\Sigma_{j=1}^{5}X_{ij}=1∼Multinomial\left(P\right). \end{array}$$

Then, the profile log likelihood is:

$$\begin{array}{c} l\left(x,P\right)=\sum_{j=1}^{5}\sum_{i=1}^{6}x_{ij}log\left(p_{j}\right). \end{array}$$

We know the observation from Fig 1 is:

$$\begin{array}{c} X=(\begin{array}{ccccc} 1 & 0 & 0 & 0 & 0\\ x_{21} & x_{22} & 0 & 0 & 0\\ x_{31} & x_{32} & x_{33} & 0 & 0\\ 0 & 0 & x_{43} & x_{44} & 0\\ 0 & 0 & x_{53} & x_{54} & x_{55}\\ 0 & 0 & 0 & 0 & 1 \end{array}). \end{array}$$ (2)

In the second short read, we know that one of *x*_21_ and *x*_22_ is 1, but we do not know which one. Therefore, we consider *x*_21_ and *x*_22_ as latent variables and estimate *P* using the EM algorithm, which is given below:

**E-step**:

Given the current estimation *P*^−^ for *P*, the conditional expectation of the log complete data likelihood is:

$$\begin{array}{ccc} Q(P|P^{-}) & = & E(l\left(P|X^{\left(obs)\right)}\right)|P^{-})\\ & = & \sum_{j=1}^{5}\sum_{i=1}^{6}{\tilde{x}}_{ij}log\left(p_{j}\right). \end{array}$$

Given this observation, E-step [12] consists of computing the following quantities:

$$\begin{array}{ccc} {\tilde{x}}_{ij} & = & E(X_{ij}|P^{-},X^{\left(obs\right)}); \end{array}$$

therefore,

$$\begin{array}{ccc} Q(P|P^{-}) & = & \left(1+\frac{p_{1}^{-}}{p_{1}^{-}+p_{2}^{-}}+\frac{p_{1}^{-}}{p_{1}^{-}+p_{2}^{-}+p_{3}^{-}}\right)log\left(p_{1}\right)\\ & + & \left(\frac{p_{2}^{-}}{p_{1}^{-}+p_{2}^{-}}+\frac{p_{2}^{-}}{p_{1}^{-}+p_{2}^{-}+p_{3}^{-}}\right)log\left(p_{2}\right)\\ & + & \left(\frac{p_{3}^{-}}{p_{1}^{-}+p_{2}^{-}+p_{3}^{-}}+\frac{p_{3}^{-}}{p_{3}^{-}+p_{4}^{-}}+\frac{p_{3}^{-}}{p_{3}^{-}+p_{4}^{-}+p_{5}^{-}}\right)log\left(p_{3}\right)\\ & + & \left(\frac{p_{4}^{-}}{p_{3}^{-}+p_{4}^{-}}+\frac{p_{4}^{-}}{p_{3}^{-}+p_{4}^{-}+p_{5}^{-}}\right)log\left(p_{4}\right)\\ & + & \left(\frac{p_{5}^{-}}{p_{3}^{-}+p_{4}^{-}+p_{5}^{-}}+1\right)log\left(p_{5}\right). \end{array}$$

**M-Step**:

During the M-step, the goal is to maximize *Q*(*P* ∣ *P*^−^) with respect to *P*, which requires solving ∂*Q*(*P* ∣ *P*^−^)/∂*P* = 0 subject to $\sum_{j=1}^{5}p_{j}=1$. That is,

$$\begin{array}{c} Q^{*}=Q\left(P|P^{-}\right)-\lambda \left(\sum_{j=1}^{5}p_{j}-1\right). \end{array}$$

Then, we solve the following equation system to obtain updated parameter estimates:

$$\begin{array}{c} \frac{\partial Q^{*}}{\partial P_{j}}=0. \end{array}$$

Therefore, the update formula of *P* changed, as follows:

$$\begin{array}{ccc} {\hat{p}}_{1} & = & (1+\frac{p_{1}^{-}}{p_{1}^{-}+p_{2}^{-}}+\frac{p_{1}^{-}}{p_{1}^{-}+p_{2}^{-}+p_{3}^{-}})/6,\\ {\hat{p}}_{2} & = & (\frac{p_{2}^{-}}{p_{1}^{-}+p_{2}^{-}}+\frac{p_{2}^{-}}{p_{1}^{-}+p_{2}^{-}+p_{3}^{-}})/6,\\ {\hat{p}}_{3} & = & (\frac{p_{3}^{-}}{p_{1}^{-}+p_{2}^{-}+p_{3}^{-}}+\frac{p_{3}^{-}}{p_{3}^{-}+p_{4}^{-}}+\frac{p_{3}^{-}}{p_{3}^{-}+p_{4}^{-}+p_{5}^{-}})/6,\\ {\hat{p}}_{4} & = & (\frac{p_{4}^{-}}{p_{3}^{-}+p_{4}^{-}}+\frac{p_{4}^{-}}{p_{3}^{-}+p_{4}^{-}+p_{5}^{-}})/6,\\ {\hat{p}}_{5} & = & (\frac{p_{5}^{-}}{p_{3}^{-}+p_{4}^{-}+p_{5}^{-}}+1)/6. \end{array}$$

The iteration process is the same as Ting’s algorithm when we give equal value to the starting value for $p_{i}^{\left(0\right)}$. In fact, the starting value does not affect the convergence value. Then, [6*P*] is the number of short reads at each CpG site.

### 3.2 At least a CpG contributes to a short read

If one short read covers several CpG sites, it actually comes from at least one of them, even though we do not know which CpG sites they are. Then, under the condition $\Sigma_{j=1}^{5}X_{ij}\geq 1$, the distribution of *X*_*i*_ = (*X*_*i*1_, *X*_*i*2_, *X*_*i*3_, *X*_*i*4_, *X*_*i*5_) is:

$$\begin{array}{ccc} & & p(X_{i1},\;X_{i2},\;X_{i3},\;X_{i4},\;X_{i5}|\Sigma_{j=1}^{5}X_{ij}\geq 1),\\ & = & \frac{p\left(X_{i1},\;X_{i2},\;X_{i3},\;X_{i4},\;X_{i5}\right)-p\left(X_{i1},\;X_{i2},\;X_{i3},\;X_{i4},\;X_{i5},\;\Sigma_{j=1}^{5}X_{ij}=0\right)}{p(\Sigma_{j=1}^{5}X_{ij}\geq 1)}\\ & = & \frac{\prod_{j=1}^{5}q_{j}^{X_{ij}}{\left(1-q_{j}\right)}^{1-X_{ij}}}{1-\prod_{j=1}^{5}\left(1-q_{j}\right)}, \end{array}$$

where *q*_*j*_ is defined in formula (1).

We know the observation is expressed in the matrix as (2). In the second read, we know that some of *x* are indeterminate. Therefore, we consider the missed values of *x* as latent variables and estimate *q* = (*q*_1_, ⋯*q*_5_) using the EM algorithm.

**E-step**:

Given the current estimation *q*^−^ for *q*, the conditional expectation of the log complete data likelihood is given as:

$$\begin{array}{ccc} Q(q|q^{-}) & = & E(l\left(q|x^{\left(obs)\right)}\right)|q^{-})\\ & = & \sum_{j=1}^{5}\sum_{i=1}^{6}{\tilde{x}}_{ij}log\left(q_{j}\right)-6log\left(1-\Pi_{j=1}^{5}\left(1-q_{j}\right)\right), \end{array}$$

where ${\tilde{x}}_{ij}$ is replaced by the condition expectation.

$$\begin{array}{ccc} {\tilde{x}}_{ij} & = & E(X_{ij}|q^{-},x^{\left(obs\right)}), \end{array}$$

for example, ${\tilde{x}}_{21}=E(X_{21}|P^{-},x_{23}=0,x_{24}=0,x_{25}=0)=\frac{q_{1}^{-}}{1-\Pi_{j=1}^{2}(1-q_{j}^{-})}$.

Therefore,

$$\begin{array}{ccc} Q(P|P^{-}) & = & \left(1+\frac{q_{1}^{-}}{1-\Pi_{j=1}^{2}\left(1-q_{j}^{-}\right)}+\frac{q_{1}^{-}}{1-\Pi_{j=1}^{3}\left(1-q_{j}^{-}\right)}\right)log\left(q_{1}\right)\\ & + & \left(\frac{q_{2}^{-}}{1-\Pi_{j=1}^{2}\left(1-q_{j}^{-}\right)}+\frac{q_{2}^{-}}{1-\Pi_{j=1}^{3}\left(1-q_{j}^{-}\right)}\right)log\left(q_{2}\right)\\ & + & \left(\frac{q_{3}^{-}}{1-\Pi_{j=1}^{3}\left(1-q_{j}^{-}\right)}+\frac{q_{3}^{-}}{1-\Pi_{j=3}^{4}\left(1-q_{j}^{-}\right)}+\frac{q_{3}^{-}}{1-\Pi_{j=3}^{5}\left(1-q_{j}^{-}\right)}\right)log\left(q_{3}\right)\\ & + & \left(\frac{q_{4}^{-}}{1-\Pi_{j=3}^{4}\left(1-q_{j}^{-}\right)}+\frac{q_{4}^{-}}{1-\Pi_{j=3}^{5}\left(1-q_{j}^{-}\right)}\right)log\left(q_{4}\right)\\ & + & \left(\frac{q_{5}^{-}}{1-\Pi_{j=3}^{5}\left(1-q_{j}^{-}\right)}+1\right)log\left(q_{5}\right)-6log\left(1-\Pi_{j=1}^{5}\left(1-q_{j}\right)\right). \end{array}$$

**M-Step**:

During the M-step, the goal is to maximize *Q*(*q* ∣ *q*^−^) with respect to *q*, which requires solving ∂*Q*(*q* ∣ *q*^−^)/∂*q* = 0. That is,

$$\begin{array}{c} Q^{*}=Q\left(q|q^{-}\right)-\lambda \left(\sum_{j=1}^{5}q_{j}-1\right). \end{array}$$

Then, we solve the following equation system to obtain updated parameter estimates:

$$\begin{array}{c} \frac{\partial Q^{*}}{\partial q_{j}}=0. \end{array}$$

Therefore, the update formula of *q* is changed, as follows:

$$\begin{array}{ccc} {\hat{q}}_{1} & = & \left(1+\frac{q_{1}^{-}}{1-\Pi_{j=1}^{2}\left(1-q_{j}^{-}\right)}+\frac{q_{1}^{-}}{1-\Pi_{j=1}^{3}\left(1-q_{j}^{-}\right)}\right)\left(1-\Pi_{j=1}^{5}\left(1-q_{j}^{-}\right)\right)/6,\\ {\hat{q}}_{2} & = & \left(\frac{q_{2}^{-}}{1-\Pi_{j=1}^{2}\left(1-q_{j}^{-}\right)}+\frac{q_{2}^{-}}{1-\Pi_{j=1}^{3}\left(1-q_{j}^{-}\right)}\right)\left(1-\Pi_{j=1}^{5}\left(1-q_{j}^{-}\right)\right)/6,\\ {\hat{q}}_{3} & = & \left(\frac{q_{3}^{-}}{1-\Pi_{j=1}^{3}\left(1-q_{j}^{-}\right)}+\frac{q_{3}^{-}}{1-\Pi_{j=3}^{4}\left(1-q_{j}^{-}\right)}+\frac{q_{3}^{-}}{1-\Pi_{j=3}^{5}\left(1-q_{j}^{-}\right)}\right)\left(1-\Pi_{j=1}^{5}\left(1-q_{j}^{-}\right)\right)/6,\\ {\hat{q}}_{4} & = & \left(\frac{q_{4}^{-}}{1-\Pi_{j=3}^{4}\left(1-q_{j}^{-}\right)}+\frac{q_{4}^{-}}{1-\Pi_{j=3}^{5}\left(1-q_{j}^{-}\right)}\right)\left(1-\Pi_{j=1}^{5}\left(1-q_{j}^{-}\right)\right)/6,\\ {\hat{q}}_{5} & = & \left(\frac{q_{5}^{-}}{1-\Pi_{j=3}^{5}\left(1-q_{j}^{-}\right)}+1\right)\left(1-\Pi_{j=1}^{5}\left(1-q_{j}^{-}\right)\right)/6. \end{array}$$

The starting value does not affect the convergence value. Then, $\left[6q/(1-\Pi_{j=1}^{5}(1-q_{j}))\right]$ is the number of short reads at each CpG site.

## 4 Real data analysis

To evaluate the performance of the proposed method, we compare it with the existing method (Raw) that directly uses the observation fragments. The data comes from paper [11], which includes 19 human samples. In this paper, we only consider two samples, embryonic stem cells (the ES cell line H1) and human fetal neural stem cells (NSCs) culture (HuFNSC02, neurosphere cultured cells, ganglionic eminence derived, fetal age of 21 weeks). Then, we obtained the MeDIP-seq and MRE-seq data for each sample. Similar to the analysis procedure for the M&M method, we test the performance of SIMD and the existing method by pair-wise comparisons between two H1-ESC replicates and between H1-ESCs and fetal NSCs. The difference between the two tests is that we detect differentially methylated CpG sites in this paper; however, the test of the M&M method is to determine differentially methylated regions (DMRs).

We consider the false positive rates for two methods. We apply SIMD and the existing method to the two H1-ESC replicates and use the hypothesis test to obtain the *P*-values for each CpG site. Because the H1-ESC samples are biological replicates, we consider the different methylated CpG sites as the false discovery sites at any *P*-value cutoff. The results are represented in Table 1. It is evident from the table that at the same *P*-value cutoff, SIMD usually reports fewer differentially methylated CpG sites than the exisiting method; for example, when the *P*-value cutoff equals 10^−5^, the number of differentially methylated CpG sites for the existing method is seven times more than for SIMD. There are approximately 1751273 CpG sites in chromosome 1 of the human reference sequence (excluding blacklist regions). We can then calculate the false positive rates for two methods at any *P*-value cutoff. Obviously, from Table 1, we can see that false positive rates of SIMD are significantly less than those of the existing method.

**Table 1:** The false positive numbers of two methods at each *p*-value cutoff (two H1-ESCs).

Levels	1e-3	1e-4	1e-5	1e-6	1e-7	1e-8
False positive numbers						
SIMD	3621	1054	295	115	45	33
Raw	13336	4915	2089	1047	607	415

Next, we consider the false discovery rates(FDRs) for two methods. We compare two different cell types, H1-ESC and fetal NSCs, and use the same *P*-value cutoffs as the first test. We obtain the number of differentially methylated CpG sites for two methods. Combining the results of the two H1-ESC replicates for analysis, we obtain the false discovery rates at any *P*-value cutoff. From Table 2, we can see that the number and FDRs of SIMD are no better than the existing method when the cutoffs are larger than 10^−6^. However, when cutoffs are smaller than 10^−6^, the FDRs of SIMD are obviously less than those of the existing method. Next, we further consider the *q*-value cutoffs in Table 3, similar to the *P*-value cutoff, and find that the number of differentially methylated CpG sites of SIMD is far less than in the method at each *q*-value level (approximately 1/20 of the existing method). However, the FDRs of the existing method are larger than the overall SIMD.

**Table 2:** The differentially methylated site number of two methods at each *p*-value cutoff (chr1 of H1 vs HuFNSC02).

Levels	1e-3	1e-4	1e-5	1e-6	1e-7	1e-8
Differentially methylated CpG sites number						
SIMD	7830	2518	838	395	198	135
Raw	31653	12796	5997	3304	2110	1474
FDRs of two methods						
SIMD	0.46245	0.41858	0.35202	0.29113	0.22727	0.24444
Raw	0.42131	0.38410	0.34834	0.31688	0.28767	0.28154

**Table 3:** The number of differentially methylated sites derived from two methods at each *q*-value cutoff (chr1 of H1 vs HuFNSC02).

Levels	5e-2	1e-2	1e-3	1e-4	1e-5	1e-6
Differentially methylated CpG sites number						
SIMD	1259	542	199	105	67	33
Raw	27412	11106	4070	2100	1363	885
FDRs of two methods						
SIMD	0.36536	0.31549	0.22110	0.22857	0.17910	0.2121
Raw	0.41853	0.38015	0.32776	0.29142	0.27953	0.2655

## 5 Discussion

Identifying differentially methylated CpG sites across a whole genome is an effective way to study epigenetic modification. In dealing with the data integrated by MeDIP-seq and MRE-seq, estimating the methylation level is the first choice. In this paper, we proposed a SIMD method that considers the possible structure whereby immunoprecipitated short reads are mapped to the methylated CpG sites. We then proposed two cases based on it, one in which only a single CpG site contributes to a short read and another in which more than one CpG site contributes to a short read. By applying the SIMD method, we can obtain the real number of short reads in each CpG site. Last, we employ the hypothesis test framework to detect the differentially methylated CpG sites.

In real data analysis, we compare the proposed SIMD method with the existing method(Raw). The results demonstrate that although the number of differentially methylated CpG sites detected by the SIMD method is less than those detected by the existing method, the FDRs of the SIMD are much smaller than those of the existing method. The conclusion is that the proposed method performs better than the existing method. There are still some problems, such as the assumption of independence between the short reads. When the independence condition cannot be satisfied, the proposed method may work not very well. Therefore, in our future work, we will take the correlation between the short reads that are mapped to the neighboring CpG sites into account.

### Appendix A: Proof of Theorem 1

Under Assumption 1, the joint distribution of (*X*_*i*1_, *X*_*i*2_, ⋯, *X*_*ij*_, ⋯, *X*_*iG*_) is

$$\begin{array}{c} P\left(X_{i1},\;X_{i2},\;\cdots ,X_{ij},\;\cdots ,\;X_{iG}\right)=\prod_{j=1}^{G}{\left(\frac{\lambda_{j}}{1+\lambda_{j}}\right)}^{X_{ij}}{\left(\frac{1}{1+\lambda_{j}}\right)}^{1-X_{ij}}, \end{array}$$

then,

$$\begin{array}{ccc} & & P(X_{i1},\;X_{i2},\;\cdots ,X_{ij},\;\cdots ,\;X_{iG}|\Sigma_{j=1}^{G}X_{ij}=1)\\ & = & \frac{P(X_{i1},\;X_{i2},\;\cdots ,X_{ij},\;\cdots ,\;X_{iG},\Sigma_{j=1}^{G}X_{ij}=1)}{P(\Sigma_{j=1}^{G}X_{ij}=1)}\\ & = & \frac{\prod_{j=1}^{\left(G-1\right)}{\left(\frac{\lambda_{j}}{1+\lambda_{j}}\right)}^{X_{ij}}{\left(\frac{1}{1+\lambda_{j}}\right)}^{1-X_{ij}}{\left(\frac{\lambda_{G}}{1+\lambda_{G}}\right)}^{\left(1-\sum_{j=1}^{\left(G-1\right)}X_{ij}\right)}{\left(\frac{1}{1+\lambda_{G}}\right)}^{\sum_{j=1}^{\left(G-1\right)}X_{ij})}}{\sum_{j=1}^{G}\lambda_{j}}\\ & = & \frac{\prod_{j=1}^{\left(G-1\right)}\lambda_{j}^{X_{ij}}\lambda_{G}^{\left(1-\sum_{j=1}^{\left(G-1\right)}X_{ij}\right)}}{\sum_{j=1}^{G}\lambda_{j}}\\ & = & \prod_{j=1}^{\left(G-1\right)}{\left(\frac{\lambda_{j}}{\sum_{j=1}^{G}\lambda_{j}}\right)}^{X_{ij}}{\left(\frac{\lambda_{G}}{\sum_{j=1}^{G}\lambda_{j}}\right)}^{\left(1-\sum_{j=1}^{\left(G-1\right)}X_{ij}\right)}. \end{array}$$

This is the end of the proof.

### Appendix B: Proof of Theorem 2

$$\begin{array}{ccc} & & P(X_{i1},\;X_{i2},\;\cdots ,X_{ij},\;\cdots ,\;X_{iG}|\Sigma_{j=1}^{G}X_{ij}\geq 1)\\ & = & \frac{P(X_{i1},\;X_{i2},\;\cdots ,X_{ij},\;\cdots ,\;X_{iG},\Sigma_{j=1}^{G}X_{ij}\geq 1)}{P(\Sigma_{j=1}^{G}X_{ij}\geq 1)}\\ & = & \frac{P\left(X_{i1},\;X_{i2},\;\cdots ,X_{ij},\;\cdots ,\;X_{iG}\right)-P\left(X_{i1},\;X_{i2},\;\cdots ,X_{ij},\;\cdots ,\;X_{iG},\Sigma_{j=1}^{G}X_{ij}=0\right)}{1-P(\Sigma_{j=1}^{G}X_{ij}=0)}\\ & = & \frac{\prod_{j=1}^{G}q_{j}^{X_{ij}}{\left(1-q_{j}\right)}^{1-X_{ij}}}{1-\prod_{j=1}^{G}\left(1-q_{j}\right)}, \end{array}$$

where $P(X_{i1},\;X_{i2},\;\cdots ,X_{ij},\;\cdots ,\;X_{iG}|\Sigma_{j=1}^{G}X_{ij}=0)=0$ in real data. This is the end of the proof.

### Appendix C: EM algorithm for Theorems 1 and 2

We know the observation is

$$\begin{array}{c} X=(\begin{array}{ccccccccc} 0 & \cdots & 0 & x_{1k_{1}} & \cdots & x_{1l_{1}} & 0 & \cdots & 0\\ 0 & \cdots & 0 & x_{2k_{2}} & \cdots & x_{2l_{2}} & 0 & \cdots & 0\\ \cdot & \cdots & \cdot & \cdot & \cdots & \cdot & \cdot & \cdots & \cdot \\ 0 & \cdots & 0 & x_{ik_{i}} & \cdots & x_{il_{i}} & 0 & \cdots & 0\\ \cdot & \cdots & \cdot & \cdot & \cdots & \cdot & \cdot & \cdots & \cdot \\ 0 & \cdots & 0 & x_{nk_{n}} & \cdots & x_{nl_{n}} & 0 & \cdots & 0 \end{array}), \end{array}$$

where $x_{ik_{i}},\cdots ,x_{il_{i}}$ are missed data. However, we know some of $x_{ik_{i}},\cdots ,x_{il_{i}}$ are 1 and others are 0. Therefore, the observation of each read is $x^{\left(obs\right)}=(x_{1}^{\left(obs\right)},x_{2}^{\left(obs\right)},\cdots ,x_{n}^{\left(obs\right)})$, where $x_{i}^{\left(obs\right)}=(x_{i1}=0,\cdots ,x_{i(k_{i}-1)}=0,x_{i(l_{i}+1)}=0,\cdots ,x_{iG}=0)$. There are two cases.

(1) Only a single CpG contributes to a short read:

If one short read covers several CpG sites, it actually only comes from one of them, even though we do not know which one it is. That is, we have known that $\Sigma_{j=1}^{G}X_{ij}=1$, the joint distribution of *X*_*i*1_, ⋯, and *X*_*iG*_ is a multinomial distribution with probability *P* = (*p*_1_, ⋯, *p*_*G*_), where $p_{j}=\lambda_{j}/\Sigma_{j=1}^{G}\lambda_{j}$. A short read is an observation that is *X*_*i*_ = (*x*_*i*1_, *x*_*i*2_, ⋯, *x*_*iG*_), where *x*_*ij*_ = 0 or 1. A note is that only one element of *X*_*i*_ is 1 and the others are 0. The *x*_*ij*_ will be 0 when the *j*th CpG is not covered by the *i*th short reads. That is,

$$\begin{array}{c} X_{i}|\Sigma_{j=1}^{G}X_{ij}=1∼Multinomial\left(P\right). \end{array}$$

Then, the profile log likelihood is:

$$\begin{array}{c} l\left(x,P\right)=\sum_{j=1}^{G}\sum_{i=1}^{n}x_{ij}log\left(p_{j}\right). \end{array}$$

The EM algorithm is

**E-step**:

Given the current estimate *P*^−^ for *P*, the conditional expectation of the log complete data likelihood is given as:

$$\begin{array}{ccc} Q(P|P^{-}) & = & E(l\left(P|x^{\left(obs)\right)}\right)|P^{-})\\ & = & \sum_{j=1}^{G}\sum_{i=1}^{n}{\tilde{x}}_{ij}log\left(p_{j}\right). \end{array}$$

Given this, the E-step [13] consists of computing the following quantities:

$$\begin{array}{cc} {\tilde{x}}_{ij} & =\{\begin{array}{cc} E(X_{ij}|P^{-},x_{i}^{\left(obs\right)}) & \;\;\;k_{i}\leq j\leq l_{i};\\ 0 & \;\;\;others. \end{array} \end{array}$$

We know that the marginal distribution of $x_{ik_{i}},\cdots ,x_{il_{i}}$ is also a multinomial distribution or binomial distribution. Then ${\tilde{x}}_{ij}=\frac{p_{j}^{-}}{\sum_{s=k_{i}}^{l_{i}}p_{s}^{-}}$, where *k*_*i*_ ≤ *j* ≤ *l*_*i*_.

**M-Step**:

During the M-step, the goal is to maximize *Q*(*P* ∣ *P*^−^) with respect to *P*, which requires solving ∂*Q*(*P* ∣ *P*^−^)/∂*p* = 0 subject to $\sum_{j=1}^{G}p_{j}=1$. That is,

$$\begin{array}{c} Q^{*}=Q\left(P|P^{-}\right)-\lambda \left(\sum_{j=1}^{G}p_{j}-1\right). \end{array}$$

Then, we solve the following equation system to obtain updated parameter estimates:

$$\begin{array}{c} \frac{\partial Q^{*}}{\partial P_{j}}=0. \end{array}$$

Thus, given below is the updated formula of *P*:

$$\begin{array}{ccc} {\hat{p}}_{j} & = & \sum_{i=1}^{n}{\tilde{x}}_{ij}\left(P^{-}\right)/n. \end{array}$$

The iteration process is the same as Ting’s algorithm when we give equal value to the starting value for $p_{i}^{\left(0\right)}$. In fact, the starting value does not affect the convergence value. Then, [*nP*] is the number of short reads at each CpG site.

(2) The case that at least a CpG contributes to a short read:

The process of the proof is the same as (1).
